# Polo-like Kinase 1 Activation Regulates Angiotensin II-Induced Contraction in Pudendal and Small Mesenteric Arteries from Mice

**DOI:** 10.3390/cells14211741

**Published:** 2025-11-06

**Authors:** Raiana Anjos Moraes, Olufunke O. Arishe, James Pratt, Stephanie Wilczynski, Rinaldo Rodrigues dos Passos, Diana L. Silva-Velasco, Tiago Tomazini Gonçalves, Tianxin Zhang, Darizy Flavia Silva, R. Clinton Webb, Fernanda Priviero

**Affiliations:** 1 Cardiovascular Translational Research Center, University of South Carolina, Columbia, SC 29209, USA; james.pratt@uscmed.sc.edu (J.P.); stephanie.wilczynski@uscmed.sc.edu (S.W.); rinaldo.rodrigues@uscmed.sc.edu (R.R.d.P.); diana.silvavelasco@uscmed.sc.edu (D.L.S.-V.); tiago.tomazinigoncalves@uscmed.sc.edu (T.T.G.); tianxin.zhang.viatrix@gmail.com (T.Z.); clinton.webb@uscmed.sc.edu (R.C.W.); fernanda.priviero@uscmed.sc.edu (F.P.); 2 Department of Cell Biology and Anatomy, School of Medicine, University of South Carolina, Columbia, SC 29209, USA; 3 Department of Medical Pharmacology and Physiology, University of Missouri, Columbia, MO 65212, USA; oarishe@missouri.edu; 4 Laboratory of Cardiovascular Physiology and Pharmacology, Federal University of Bahia, Salvador 40110-907, BA, Brazil; darizy@gmail.com; 5 Department of Biomedical Engineering, University of South Carolina, Columbia, SC 29209, USA

**Keywords:** polo-like kinase 1, contraction, smooth muscle cells, angiotensin II

## Abstract

Polo-like kinase 1 (PLK1) is a serine/threonine protein kinase expressed in smooth muscle cells (SMCs), with emerging roles in regulating contraction. We hypothesize that PLK1 contributes to smooth muscle contractility in pudendal arteries (PA), small mesenteric arteries (SMA), and the corpus cavernosum (CC). Using male C57BL/6J mice, we assessed mRNA and protein expression of PLK1 in these tissues. In addition, the arteries and CC were mounted in myographs for isometric force measurement. We then investigated whether PLK1 regulates SMC contractility induced by phenylephrine (PE), U46619, and angiotensin II (Ang II) in arteries, and by PE, serotonin (5-HT), and electrical field stimulation (EFS; 1–16 Hz) in the CC, both in the presence and absence of the PLK1 inhibitor volasertib. PLK1 expression was confirmed in the SMA, PA, and CC by RT-qPCR or Western blotting. Notably, PLK1 inhibition significantly reduced Ang II-induced contraction in the PA and SMA and attenuated EFS-induced contraction at 2 and 4 Hz in the CC. In contrast, responses to PE, U46619, and 5-HT were unaffected by PLK1 inhibition. These results suggest that PLK1 selectively mediates contraction in response to Ang II and neurogenic stimuli. PLK1 may therefore represent a novel, stimulus-specific regulator of vascular and erectile smooth muscle contractility.

## 1. Introduction

Polo-like kinases (PLKs) are a family of serine/threonine kinases (PLK1–PLK5) [1]. Among these, PLK1 is the most extensively studied and is characterized by a kinase domain and a regulatory substrate-binding region known as the polo-box domain [2]. PLK1 plays essential roles in several cellular processes, including mitotic entry, centrosome maturation and separation, chromosome alignment, and cytokinesis [3,4,5]. Post-translational modifications, such as phosphorylation at Thr-210, are known to regulate PLK1 activation [6,7].

PLK1 is expressed in various tissues, including the placenta, testis, ovary [8], bronchi [7], prostate [9] and pulmonary [10] and coronary arteries [11]. Upregulation of PLK1 has been implicated in multiple cancers [12,13,14,15], making PLK1 a promising target for anticancer therapies [1,16]. Several PLK1 inhibitors have been evaluated in clinical trials, either alone or in combination with other agents [17]. For example, volasertib (BI 6727), a potent and selective PLK1 inhibitor targeting the ATP-binding pocket, has demonstrated significant antitumor activity [16,18].

Beyond its role in cancer, PLK1 has been identified as an important regulator of smooth muscle contraction [7,19]. In airway smooth muscle, PLK1 promotes contraction by phosphorylating vimentin at Ser-56 in response to contractile stimuli, thereby modulating intracellular and intercellular force transduction. Inhibition of PLK1 by volasertib relaxes precontracted tracheal rings, whereas PLK1 knockdown reduces force development, highlighting its non-mitotic role in smooth muscle contraction [7].

In vascular smooth muscle cells (SMC), PLK1 also regulates RhoA activation and actomyosin dynamics independent of its mitotic functions. It plays a crucial role in arterial structural organization, vascular tone regulation, and stiffness. Smooth muscle–specific PLK1 deletion reduces arterial elasticity, causes hypotension, and attenuates angiotensin II (Ang II)-induced contraction in aortic and mesenteric resistance artery (MRA) rings [19]. These findings indicate that PLK1 contributes to vascular homeostasis and contractile regulation.

Although PLK1 has been implicated in vascular regulation, its role in tissues critical for erectile function remains unknown. Penile erection relies on the relaxation of cavernous SMC, driven by central nervous system activation during sexual stimulation. Blood supply to erectile tissue is primarily provided by the pudendal artery (PA), which branches from the internal iliac artery [20]. Interestingly, approximately 70% of the blood flow resistance to the penis occurs in the PA, with only 30% located in intrapenile tissues [21]. Successful penile erection requires both cavernous SMC relaxation and dilation of penile arteries to ensure adequate arterial inflow into the cavernous sinusoids. Insufficient penile arterial inflow is recognized as a major cause of erectile dysfunction [22]. Previous studies have demonstrated PLK1 involvement in Ang II-induced contraction in the aorta and MRA, using BI2536 [19]. Despite these findings, the contribution of PLK1 to vascular and corpus cavernosum (CC) contraction remains poorly understood, and further research is needed to elucidate its broader role in other arteries and erectile tissues.

To address this gap, we investigated the role of PLK1 in contractile responses of the small mesenteric artery (SMA), pudendal artery (PA), and corpus cavernosum (CC) to various agonists and electrical field stimulation (EFS). We hypothesize that agonist- and neurogenic-induced contractions in these tissues are dependent on PLK1 activation, providing new insights into its role in vascular and erectile smooth muscle function.

## 2. Materials and Methods

### 2.1. Animals

The Institutional Animal Care and Use Committee at the University of South Carolina examined and granted approval for all animal procedures (number 101687). Every animal procedure was conducted in accordance with the National Institutes of Health’s (NIH) Guide for the Care and Use of Laboratory Animals. Male wild-type mice (C57BL/6J; 12–16 weeks old) were obtained from Jackson Laboratory (Bar Harbor, ME, USA), which were kept in housing with 12 h light/dark cycles, free access to a standard chow diet, and unlimited access to water. Under isoflurane anesthesia (5% in 100% O_2_), animals were sacrificed by bilateral thoracotomy.

### 2.2. PLK1 Expression

For PLK1 expression analysis, PA, CC, and SMA tissues were used for RT-qPCR, whereas CC and SMA were also analyzed by Western blotting. Because SMA and CC yielded more tissue, each was divided into two equal portions for both assays. In contrast, the limited amount of PA tissue required pooling, making it unfeasible to perform both analyses; therefore, PA was used exclusively for RT-qPCR.

#### 2.2.1. RNA Extraction and RT-qPCR

Total RNA was extracted from testis, CC, PA, and SMA using TRIzol^TM^ Reagent (Invitrogen, Carlsbad, CA, USA) according to the manufacturer’s instructions. RNA concentration and purity were assessed via spectrophotometry. cDNA was synthesized from 500 to 1000 ng of total RNA using the High-Capacity cDNA Reverse Transcription Kit (Applied Biosystems, Foster City, CA, USA). Quantitative PCR was performed using SYBR Green Master Mix (Thermo Fisher Scientific, Waltham, MA, USA) on a Bio-Rad CFX Duet system, with 25 ng of cDNA and 250 nM of each primer per reaction. The primers used for the PLK1 gene were: PLK1-Forward-5′-CAGCAAGTGGGTGGACTATT-3′ and PLK1-Reverse-5′-AGAGAATCAGGCGTGTTGAG-3′. For 18S rRNA, the primers were: 18S rRNA-Forward-5′-CTGAGAAACGGCTACCACATC-3′ and 18S rRNA-Reverse-5′-GCCTCGAAAGAGTCCTGTATTG-3′. A melt curve analysis was performed to verify product specificity. Relative gene expression was calculated using the 2^−ΔCt^ method, normalized to 18S rRNA as the reference gene, due to the absence of a single reference condition across tissues. Values were multiplied by an arbitrary unit (AU = 10^6^) for graphical presentation.

##### Agarose Gel Electrophoresis (Qualitative Verification)

For qualitative visualization of PLK1 amplification products, the PLK1 gene and the endogenous control 18S rRNA were amplified by pre-amplification (15 cycles) followed by conventional PCR using Radiant^TM^ 2X RED Taq Master Mix (Stellar Scientific, Baltimore, MD, USA). The primers used for PLK1 were: PLK1-Forward 5′-CAGCAAGTGGGTGGACTATT-3′ and PLK1-Reverse 5′-AGAGAATCAGGCGTGTTGAG-3′. For 18S rRNA, the primers were: 18S rRNA-Forward 5′-CTGAGAAACGGCTACCACATC-3′ and 18S rRNA-Reverse 5′-GCCTCGAAAGAGTCCTGTATTG-3′. The pre-amplified product was used as the template for the final PCR, with identical cycling conditions applied to both targets. Amplicons were resolved on a 3% agarose gel, stained with SYBR^TM^ Safe DNA Gel Stain (Invitrogen, Carlsbad, CA, USA), and visualized under UV illumination. No amplification was detected in the no-template control (NTC), confirming the absence of contamination.

#### 2.2.2. Western Blotting

SMA and CC were rapidly frozen in liquid nitrogen, then homogenized using ice-cold Tissue Protein Extraction Reagent (T-PER) (Thermo Fisher Scientific, Waltham, MA, USA), supplemented with protease and phosphatase inhibitor cocktails (Roche, Basel, Switzerland). Total protein concentration in the samples was measured using the BCA Protein Assay Kit (Thermo Fisher Scientific, Waltham, MA, USA). The samples were then stored at −80 °C. A total of 30 μg of protein per sample was loaded onto a 10% polyacrylamide gel following the standard SDS-PAGE protocol for Western blotting. The proteins were transferred to a nitrocellulose membrane (Thermo Fisher Scientific, Waltham, MA, USA), and the membranes were blocked at room temperature for 30 min with a solution containing 5% non-fat dry milk, followed by 1 h with 5% BSA. The membrane was incubated overnight at 4 °C with the primary antibody PLK1 (1:1000; Abcam, Cambridge, UK, AB17057). The next day, the primary antibody was retrieved, and the membrane was washed with Tris-buffered saline with Tween^®^ 20. Subsequently, the membrane was treated with the secondary antibody for 1 h at room temperature. The protein bands were detected using an enhanced chemiluminescence system, Pierce^TM^ ECL Western Blotting Substrate (Thermo Fisher Scientific, MA, USA). The specificity of the anti-PLK1 antibody (ab17057, Abcam) has been validated by the manufacturer and further demonstrated in PLK1 knockdown studies reported in the literature [23,24,25]. ImageJ version 1.54p (NIH, Bethesda, MD, USA) was used to quantify the band densities normalized to β-actin (1:2500; ABclonal, Woburn, MA, USA, AC004). Expression levels are presented as arbitrary units (A.U.).

### 2.3. Evaluation of the Role of PLK1 in Agonist-Mediated Contractions

We investigated the effects of PLK1 activation in response to different contractile agonists in the SMA, PA, and CC. For the PA and SMA, we used phenylephrine (PE), thromboxane A2 analog (U46619), and Ang II. For the CC, we used PE, serotonin (5-HT), and electrical field stimulation (1–16 Hz).

#### 2.3.1. Vascular Studies Using SMA and PA

To maintain animal tissues alive under controlled circumstances, first- and second-order branches from the SMA and the PA were quickly removed and immersed in an ice-cold physiological solution with the following content (mM): 130 NaCl, 4.7 KCl, 1.18 KH_2_PO4, 1.18 MgSO_4_.7H_2_O, 14.9 NaHCO_3_, 5.6 Dextrose, 1.56 CaCl_2_.H_2_O, 0.026 EDTA. For isometric tension recording, the PA and SMA were meticulously dissected and cleansed to eliminate fat and connective tissue, sliced into pieces measuring 2 mm in length, and mounted in tissue chambers (model 620M; Danish MyoTechnology, Aarhus, Denmark). For arteries, two steel wires with a diameter of 40 µm were inserted into the segment lumen and mounted using the technique outlined by Mulvany and Halpern [26]. The bathing solution was maintained at 37 °C and pH of approximately 7.2 while being gassed with a 95% O_2_/5% CO_2_ mixture. In order to produce active tension, vascular segments were stretched to their ideal lumen diameter. This was calculated using the internal circumference/wall tension ratio of the segments by determining the interior circumference, L_0_, to 90% of the tension that the vessels would experience if they were subjected to an amount of pressure that is equivalent to a transmural pressure of 100 mmHg (L_100_). Following stabilization, the contractility of the vessels was evaluated by stimulating them with a high-K^+^ solution and the capacity of acetylcholine (ACh; 3 µM) to generate relaxation during phenylephrine (PE; 3 µM)-induced contraction was used to examine the viability of the endothelium. Rings with relaxation levels below 70% were deemed to lack functional endothelium and were therefore eliminated from the study.

#### 2.3.2. Corpus Cavernosal Studies

CC were quickly removed and put in a physiological solution at a very low temperature (approximately 4 °C) with the following composition (mM): 130 NaCl, 4.7 KCl, 1.18 KH_2_PO4, 1.18 MgSO_4_.7H_2_O, 14.9 NaHCO_3_, 5.6 Dextrose, 1.56 CaCl_2_.H_2_O, 0.026 EDTA. Connective and adventitial tissues were removed from the penile tissue, and from its proximal extremity toward the distal extremity of the penis, the fibrous septum dividing the corpora cavernosa was cut. To obtain two strips of CC from each animal, a slit was cut lengthwise along the penile body. For the purpose of recording isometric force, each strip was put in a muscle strip myograph chamber (model 820M; Danish MyoTechnology, Aarhus, Denmark). The bathing solution was gassed with mixture of 95% O_2_/5% CO_2_ while maintaining a 37 °C. After stabilizing at a 5 mN basal tension [27], the viability of the CC was evaluated by stimulating it with a high-K^+^ solution. ACh (1 μM) was used to test the endothelial integrity in strips pre-contracted with PE (1 μM).

#### 2.3.3. Concentration-Response Curves

In separate sets of experiments, using endothelium-intact PA and SMA, concentration-response curves to PE (1 nM to 30 μM), to the thromboxane A2 analog (U46619; 0.1 nM to 3 μM), and to angiotensin II (Ang II; 0.1 nM to 100 nM) were obtained. In the CC, we conducted concentration-response curves to PE (10 nM to 30 μM) and 5-HT (5-hydroxytryptamine; 10 nM to 30 μM). Following a 30 min incubation period with volasertib (100 nM or 1 μM), concentration-response curves to the same agonist were then repeated. Volasertib (1 mM and 0.1 mM) was solubilized in 50% DMSO. To rule out the possibility that the attenuated response observed in the presence of volasertib was due to Ang II tachyphylaxis during the second curve, we repeated the Ang II curves with the vehicle.

#### 2.3.4. Corpus Cavernosum Contractile Responses to Electrical Field Stimulation

Two platinum ring electrodes attached to a Grass S88 stimulator (Astro-Med Industrial Park, West Warwick, RI, USA) promoted electrical field stimulation in CC strips that were inserted between these electrodes. According to earlier published procedures [28], contractile responses to electrical field stimulation (1–16 Hz) were carried out at 50 V, 1 ms pulse, ten-second stimuli, and a 2 min gap between stimuli. Strips were preincubated with L-NAME (100 µM), which blocks the production of nitric oxide, and atropine (1 µM), which inhibits muscarinic receptors, in order to analyze electrical field stimulation-induced contractions. Electrical field stimulation was applied to the same strip before and after treatment with volasertib (100 nM). To ensure that repeated electrical field stimulation in the same strip did not affect tissue responsiveness, we previously conducted control experiments with two consecutive electrical field stimulation applications, with vehicle treatment in between, and observed no significant differences (Figure S1).

### 2.4. Statistical Analysis

Contractions induced by PE, U46619, Ang II and 5-HT in the presence of volasertib were analyzed as a percentage of the highest contraction that would have occurred in the absence of this inhibitor, which was considered 100% in the control condition. For all concentration–response curves, both the maximal effective concentration (E_max_) and the half-maximal effective concentration (EC_50_) were evaluated to compare the magnitude and pharmacological potency of the responses. The sensitivity of the agonists is expressed as pEC_50_, the negative logarithm of the concentration of a substance that induces 50% of the maximal effect (−log EC_50_). Data analysis was performed using GraphPad Prism Software version 10 (San Diego, CA, USA). The data were examined for normality using the Shapiro–Wilk test. If the groups met the criteria for a normal distribution, paired Student’s *t*-tests were used for statistical analysis. If the groups did not meet the normality criteria, the Wilcoxon matched-pairs signed-rank test was applied. Statistical significance was considered if the *p*-value was below 0.05. Results are expressed as the mean ± standard error of the mean (SEM) from four to seven biological replicates, each representing an independent sample obtained from a different animal.

## 3. Results

### 3.1. PLK1 mRNA and PLK1 Protein Expression

**Figure 1:**
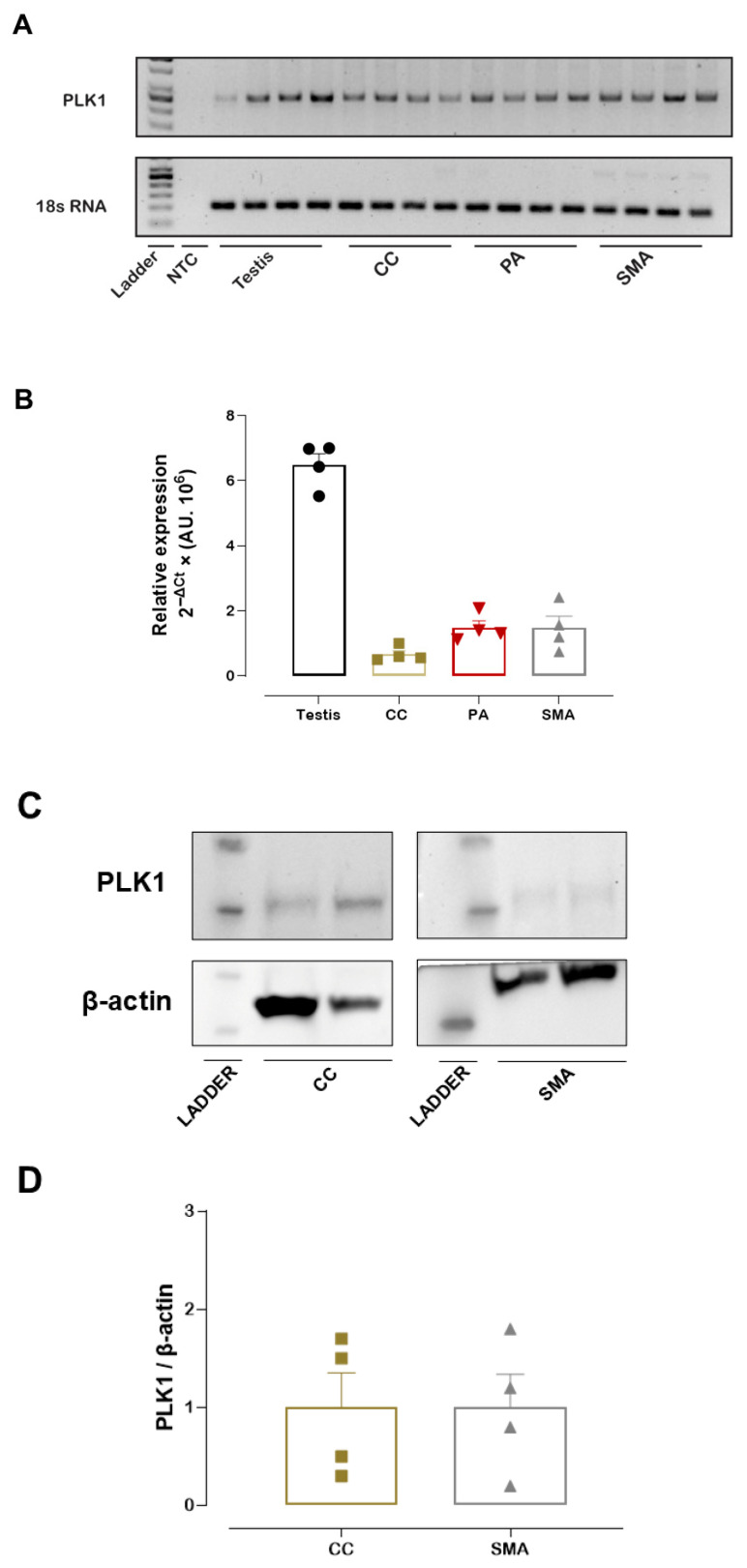
PLK1 mRNA and PLK1 protein expression. Representative agarose gel showing PLK1 and 18S rRNA amplification products in the pudendal artery (PA), corpus cavernosum (CC), and small mesenteric artery (SMA). 18S rRNA served as the internal control (**A**). Relative PLK1 mRNA expression in the PA, CC, and SMA as determined by RT-qPCR (**B**). Representative Western blot image showing PLK1 and β-actin (loading control) (**C**). Quantification of PLK1 protein expression levels in SMA and CC (**D**). Data represent the mean ± SEM.

Prior to the vascular function studies, PLK1 mRNA expression was measured in the PA, CC, and SMA, with the testis included as a positive control due to its high PLK1 expression. Both RT-qPCR analysis and a representative agarose gel confirmed that PLK1 mRNA was expressed in the PA, CC, and SMA (Figure 1A,B). Similarly, we detected PLK1 protein expression in the SMA and CC. Western blotting analysis reveals a band with an approximate molecular weight of 68 kDa, corresponding to PLK1 (Figure 1C,D). In addition, Western blotting of SMA with a different anti-PLK1 antibody (ab189139, Abcam) confirmed PLK1 expression in this tissue (Figure S2).

### 3.2. PLK1 Inhibition Did Not Affect the Contraction Induced by PE in PA and SMA

In order to investigate if PLK1 plays a role in the contraction induced by PE, experiments were performed using volasertib, a PLK1 inhibitor, using two different concentrations.

**Figure 2:**
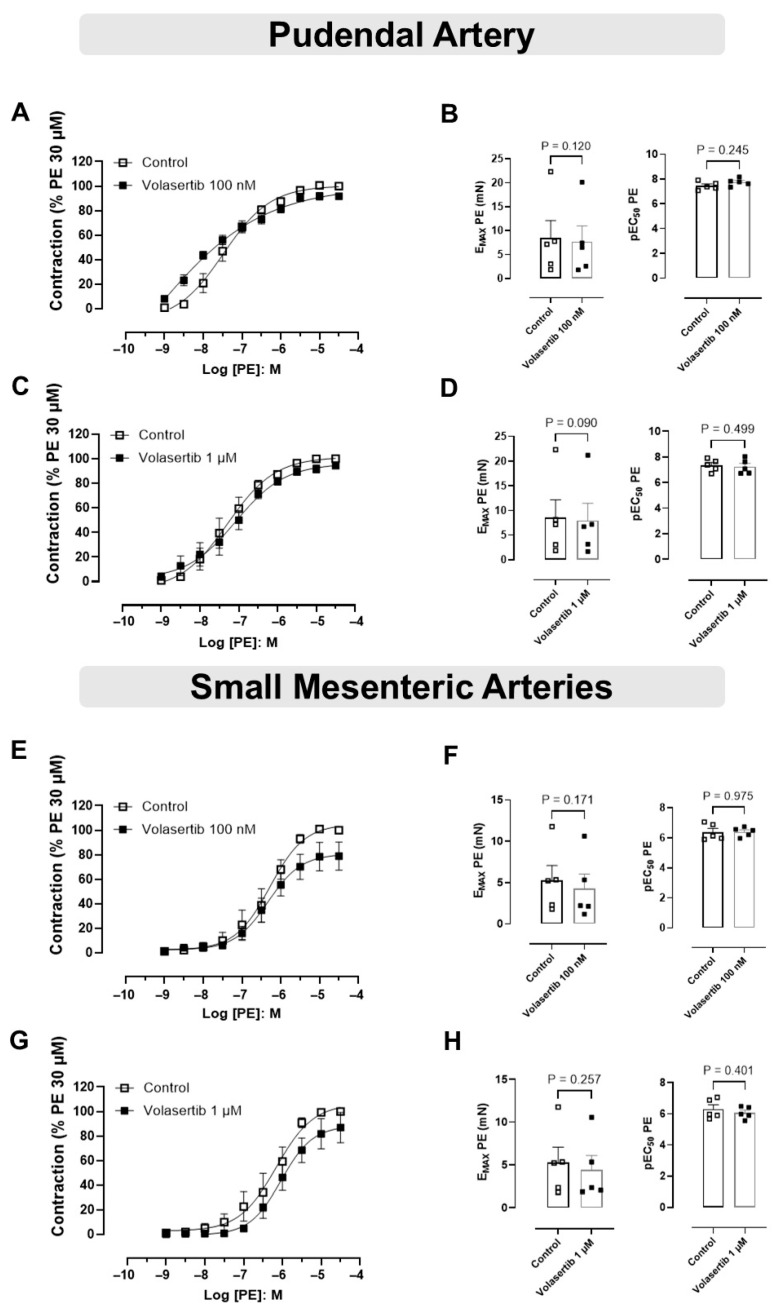
The constriction induced by PE was not influenced by PLK1 inhibitor in pudendal (PA) and small mesenteric artery (SMA). Concentration-response curves to PE (10^−9^ to 3 × 10^−5^ M) in PA (**A**,**C**) and SMA (**E**,**G**) arteries in the absence or presence of volasertib (100 nM or 1 µM). Scatter plot comparing the E_max_ and pEC_50_ (**B**,**D**,**F**,**H**) to PE in the absence or presence of volasertib (100 nM or 1 µM) on these artery rings. Data represent the mean ± SEM from 5 experiments. Statistical analysis was performed using paired Student’s *t* tests.

In PA, PLK1 inhibition by volasertib did not affect PE-induced contraction at either 100 nM or 1 µM. At 100 nM, the contraction parameters were: Control (E_max_: 8.44 ± 3.66 mN; pEC_50_: 7.46 ± 0.14) versus volasertib (E_max_: 7.69 ± 3.29 mN; pEC_50_: 7.75 ± 0.13, Figure 2A,B, n = 5). At 1 µM, the parameters were: Control (E_max_: 8.51 ± 3.65 mN; pEC_50_: 7.34 ± 0.21) versus volasertib (E_max_: 7.99 ± 3.46 mN; pEC_50_: 7.23 ± 0.26, Figure 2C,D, n = 5).

Similarly, in SMA, volasertib did not alter PE-induced contraction at either concentration. At 100 nM, the parameters were: Control (E_max_: 5.28 ± 1.78 mN; pEC_50_: 6.39 ± 0.24) versus volasertib (E_max_: 4.29 ± 1.73 mN; pEC_50_: 6.39 ± 0.14, Figure 2E,F, n = 5). At 1 µM, the parameters were: Control (E_max_: 5.29 ± 1.77 mN; pEC_50_: 6.29 ± 0.28) versus volasertib (E_max_: 4.43 ± 1.66 mN; pEC_50_: 6.07 ± 0.17, Figure 2G,H, n = 5).

These findings demonstrate that volasertib does not influence PE-induced contraction in either PA or SMA (Figure 2).

### 3.3. The Vascular Contractions Induced by U46619 Were Not Attenuated by PLK1 Inhibitor in PA and SMA

In this approach, PA and SMA were constricted using the thromboxane A2 (TX_A2_) analog U46619.

**Figure 3:**
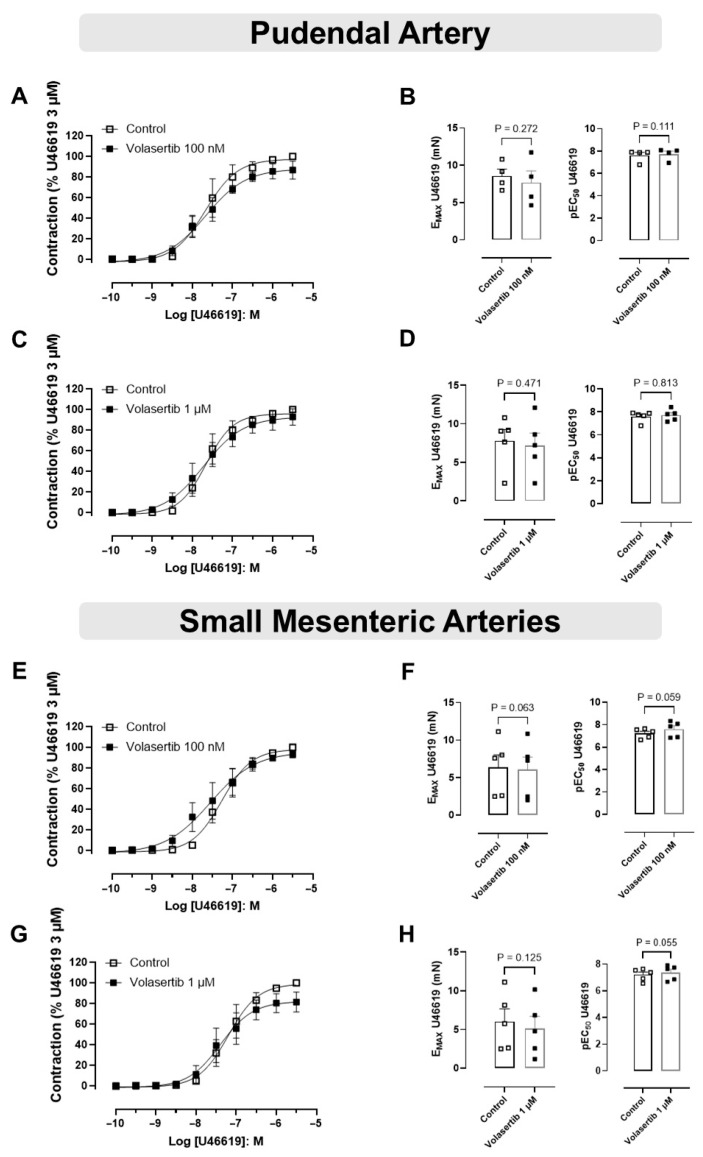
PLK1 inhibition did not affect the contraction induced by U46619 in pudendal (PA) and small mesenteric artery (SMA). Concentration-response curves to U46619 (10^−10^ to 3 × 10^−6^ M) in PA (**A**,**C**) and SMA (**E**,**G**) in the absence or presence of volasertib (100 nM or 1 µM). Scatter plot comparing the E_max_ and pEC_50_ (**B**,**D**,**F**,**H**) to U46619 in the absence or presence of volasertib (100 nM or 1 µM) on these artery rings. Data represent the mean ± SEM from 4 to 5 experiments. Statistical analysis of E_max_ (PA) and pEC_50_ (SMA) were performed using paired Student’s *t* tests. pEC_50_ (PA) and E_max_ (SMA) did not follow normal distribution; Wilcoxon test was used to determine significance. Statistical significance was considered if the *p*-value was below 0.05.

In PA, treatment with volasertib had no impact on U46619-induced contraction at either 100 nM or 1 µM concentrations. At 100 nM, the contraction parameters were: Control (E_max_: 8.57 ± 0.91 mN; pEC_50_: 7.61 ± 0.27) versus volasertib (E_max_: 7.66 ± 1.58 mN; pEC_50_: 7.73 ± 0.27, Figure 3A,B, n = 4). Similarly, at 1 µM, the parameters were: Control (E_max_: 7.80 ± 1.46 mN; pEC_50_: 7.59 ± 0.21) versus volasertib (E_max_: 7.20 ± 1.61 mN; pEC_50_: 7.71 ± 0.22, Figure 3C,D, n = 5).

In SMA, volasertib did not reduce U46619-induced contraction at either 100 nM or 1 µM concentrations. At 100 nM, the parameters were: Control (E_max_: 6.37 ± 1.67 mN; pEC_50_: 7.22 ± 0.19) versus volasertib (E_max_: 6.09 ± 1.68 mN; pEC_50_: 7.59 ± 0.31, Figure 3E,F, n = 5). At 1 µM, the parameters were: Control (E_max_: 6.02 ± 1.65 mN; pEC_50_: 7.21 ± 0.20) versus volasertib (E_max_: 5.10 ± 1.59 mN; pEC_50_: 7.37 ± 0.25, Figure 3G,H, n = 5). These results indicate that volasertib, at concentrations of 100 nM and 1 µM, did not attenuate the contraction induced by U46619.

### 3.4. PLK1 Was Involved in the Contraction Induced by Ang II in PA and SMA

To assess the role of PLK1 in Ang II-induced contraction in mice PA and SMA, we examined the effects of Ang II in the presence of volasertib.

**Figure 4:**
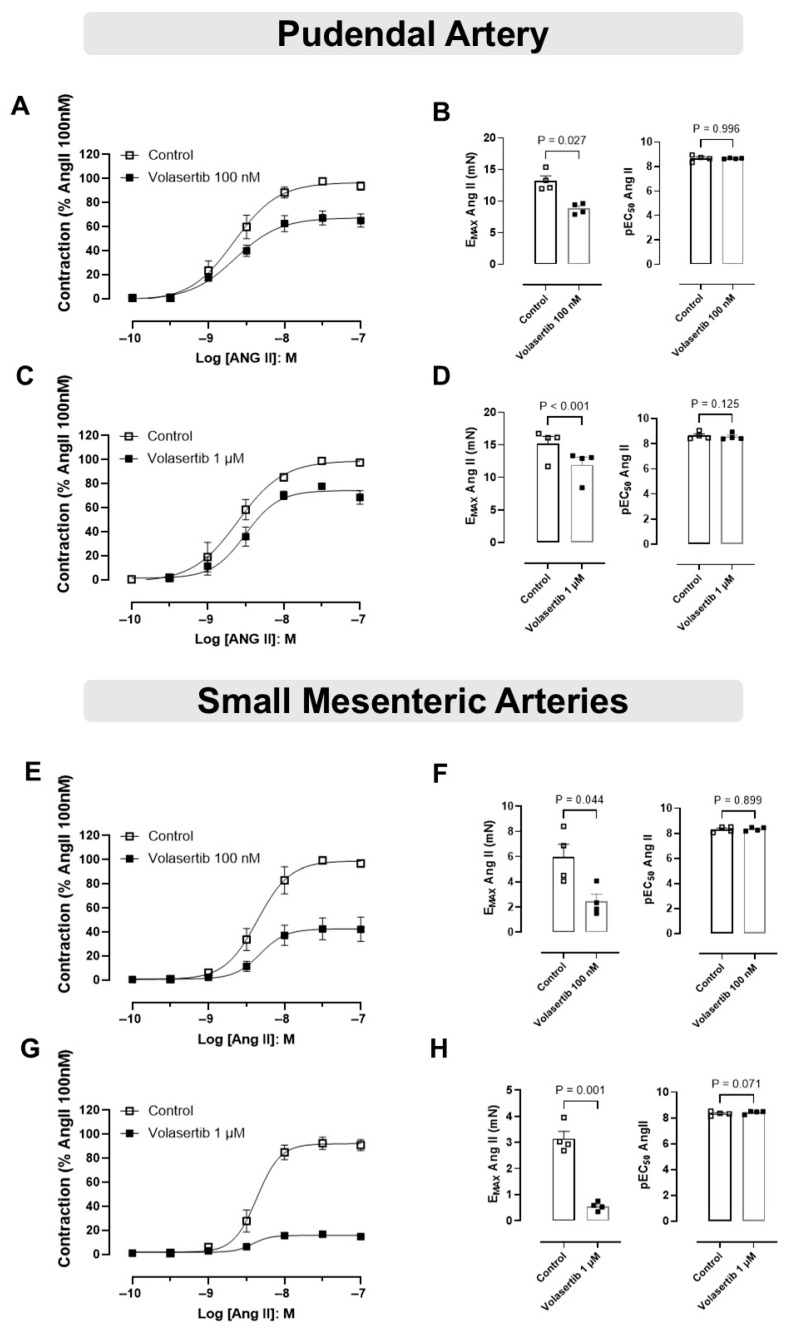
Activation of the PLK1 signaling pathway contributes to the contractile responses induced by Ang II in the pudendal (PA) and small mesenteric arteries (SMA). Concentration-response curves to Ang II (10^−10^ to 10^−7^ M) in PA (**A**,**C**) and SMA (**E**,**G**) in the absence or in the presence of volasertib (100 nM or 1 µM). Scatter plot comparing the E_max_ and pEC_50_ (**B**,**D**,**F**,**H**) to Ang II in the absence or in the presence of volasertib (100 nM or 1 µM) on these artery rings. Data represent the mean ± SEM from 4 experiments. Statistical analysis was performed using paired Student’s *t* tests. Statistical significance was considered if the *p*-value was below 0.05.

In PA, treatment with volasertib reduced Ang II-induced contraction at both 100 nM and 1 µM concentrations. At 100 nM volasertib, the contraction parameters were: Control (E_max_: 13.18 ± 0.78 mN) versus volasertib (E_max_: 8.82 ± 0.42 mN, Figure 4A,B, n = 4). Similarly, at 1 µM volasertib, the contraction parameters were: Control (E_max_: 15.15 ± 1.17 mN) versus volasertib (E_max_: 11.92 ± 1.18 mN, Figure 4C,D).

Similarly, in SMA, volasertib also reduced Ang II-induced contraction at both concentrations. At 100 nM volasertib, the parameters were: Control (E_max_: 5.96 ± 1.02 mN) versus volasertib (E_max_: 2.45 ± 0.57 mN, Figure 4E,F, n = 4). At 1 µM volasertib, the contraction parameters were: Control (E_max_: 3.15 ± 0.28 mN) versus volasertib (E_max_: 0.55 ± 0.08 mN, Figure 4G,H, n = 4).

These results suggest that at concentrations of 100 nM and 1 µM, volasertib significantly reduced Ang II-induced contractions in both PA and SMA (Figure 4).

### 3.5. The Contraction Induced by PE Was Not Changed by PLK1 Inhibition in CC

**Figure 5:**
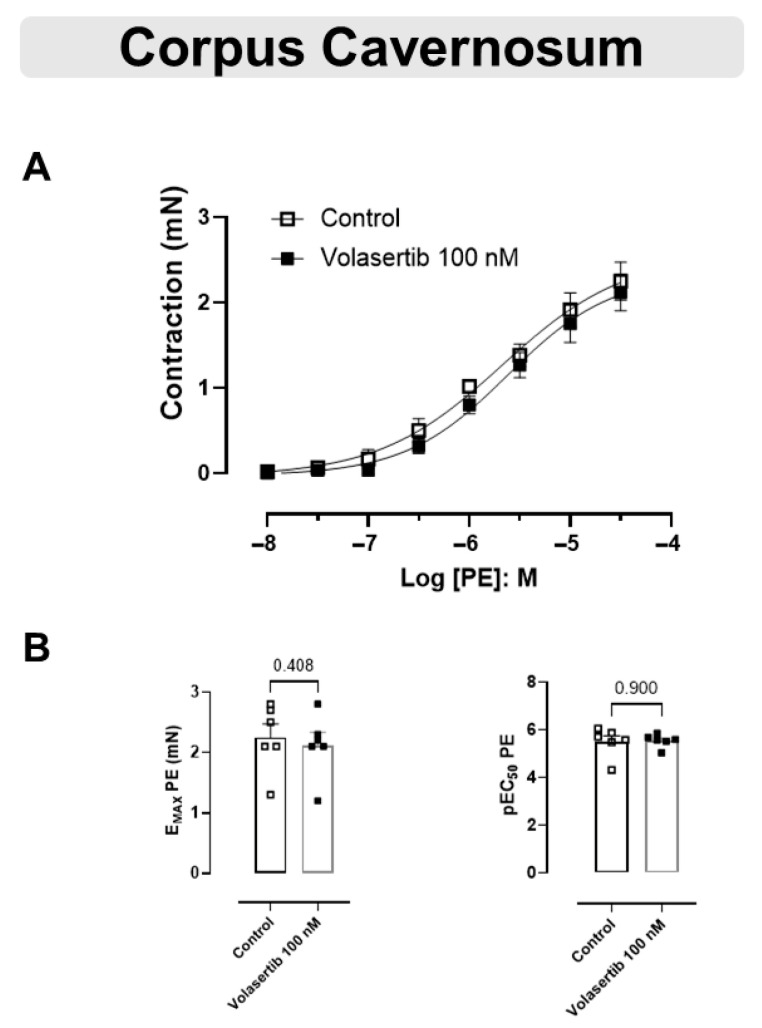
Volasertib did not alter the contraction induced by PE in CC. Concentration-response curves to PE (10^−8^ to 3 × 10^−5^ M) in CC (**A**) in the absence or presence of volasertib (100 nM). Scatter plot comparing the E_max_ and pEC_50_ (**B**) to PE in the absence or presence of volasertib (100 nM) on these cavernosal strips. Data represent the mean ± SEM from 6 experiments. Statistical analysis was performed using paired Student’s *t* tests.

These experiments used the same approach as performed previously with the arteries. Similarly, PLK1 inhibition by volasertib,100 nM, did not influence the contraction caused by PE in CC (Figure 5A,B). In CC, at 100 nM volasertib, the parameters were: Control (E_max_: 2.25 ± 0.22 mN; pEC_50_: 5.50 ± 0.25) versus volasertib (E_max_: 2.12 ± 0.21 mN; pEC_50_: 5.54 ± 0.11, n = 6).

### 3.6. PLK1 Was Not Involved in the Contraction Induced by 5-HT in CC

**Figure 6:**
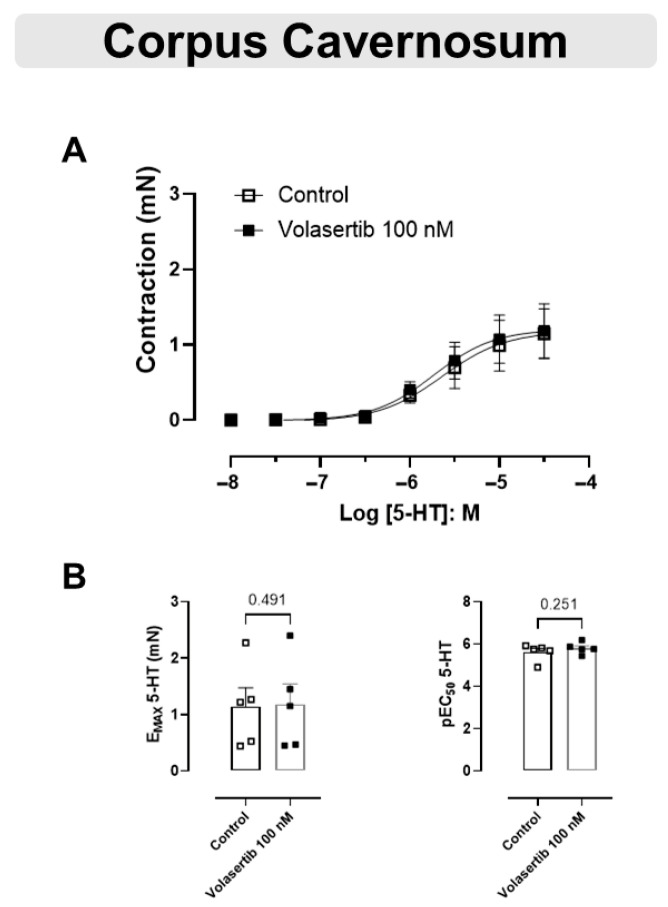
Volasertib did not change the contraction induced by 5-HT in CC. Concentration-response curves to 5-HT (10^−8^ to 3 × 10^−5^ M) in CC (**A**) in the absence or presence of volasertib (100 nM). Scatter plot comparing the E_max_ and pEC_50_ (**B**) to 5-HT in the absence or presence of volasertib (100 nM) on these cavernosal strips. Data represent the mean ± SEM from 5 experiments. Statistical analysis was performed using paired Student’s *t* tests.

The 5-HT-mediated contraction in CC was not inhibited by the PLK1 inhibitor (Figure 6A,B). In CC, at 100 nM volasertib, the parameters were: Control (E_max_: 1.14 ± 0.33 mN; pEC_50_: 5.61 ± 0.18) versus volasertib (E_max_: 1.18 ± 0.36 mN; pEC_50_: 5.80 ± 0.12, n = 5).

### 3.7. PLK1 Inhibition in CC Attenuated the Contractile Responses to Electrical Field Stimulation at 2 and 4 Hz

**Figure 7:**
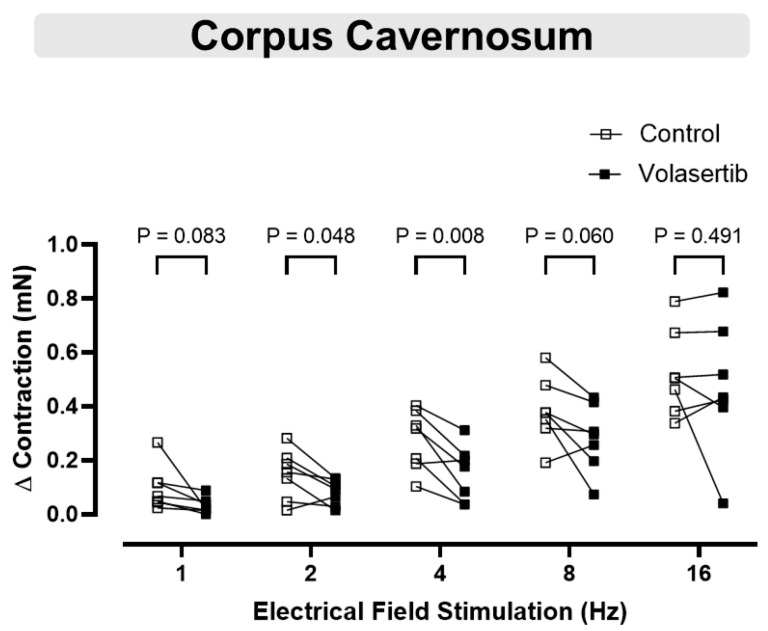
Contractile responses to electrical field stimulation at 2 and 4 Hz were reduced by PLK1 inhibition in CC. Contractile responses to electrical field stimulation (1–16 Hz) were measured in mouse corpus cavernosum before and after incubation with volasertib (100 nM). Data are expressed as mean ± SEM from 7 experiments. Statistical analysis was performed using paired Student’s *t*-tests for each frequency. Statistical significance was considered if the *p*-value was below 0.05.

Electrical field stimulation induced frequency-dependent contractions in cavernosal tissues (1–16 Hz). To investigate the involvement of PLK1 in these contractions, volasertib (100 nM) was added to the bath. Volasertib reduced the contractions induced by electrical field stimulation in CC at 2 and 4 Hz (Figure 7), suggesting that PLK1 plays a role in this process. In CC, at 1 Hz, the parameters were: Control (∆ contraction: 0.09 ± 0.03 mN) versus volasertib (∆ contraction: 0.03 ± 0.01 mN; n = 7). At 2 Hz, the parameters were: Control (∆ contraction: 0.15 ± 0.03 mN) versus volasertib (∆ contraction: 0.08 ± 0.02 mN; n = 7). At 4 Hz, the parameters were: Control (∆ contraction: 0.28 ± 0.04 mN) versus volasertib (∆ contraction: 0.15 ± 0.04 mN; n = 7). At 8 Hz, the parameters were: Control (∆ contraction: 0.38 ± 0.05 mN) versus volasertib (∆ contraction: 0.28 ± 0.05 mN; n = 7). At 16 Hz, the parameters were: Control (∆ contraction: 0.52 ± 0.06 mN) versus volasertib (∆ contraction: 0.47 ± 0.09 mN; n = 7).

## 4. Discussion

Several studies have reported a relationship between vascular dysfunction and increased vasoconstriction [29], as well as erectile dysfunction associated with enhanced contraction in the CC [30,31,32]. PLK1 is a protein kinase known for its role in cell regulation, but recent evidence also implicates it in other cellular processes, such as vascular function [19]. However, whether PLK1 contributes to smooth muscle contraction in the PA and CC has not yet been investigated.

Therefore, the present study was designed to determine the role of PLK1 in the contractile responses of the SMA, PA, and CC to various classes of agonists, as well as to electrical field stimulation in the CC, using the PLK1 inhibitor volasertib as a pharmacological tool. To the best of our knowledge, this study is the first to demonstrate PLK1 expression in the SMA, PA, and CC, and to reveal its functional involvement in Ang II-induced contraction of the PA and in electrical field stimulation-induced contractions of the CC at low stimulation frequencies (2 and 4 Hz).

PLK1 expression in these tissues was confirmed by RT-qPCR and Western blot analyses, with immunoblotting showing a band at approximately 68 kDa, corresponding to the expected molecular weight of PLK1. Based on these findings, vascular and CC experiments were performed to elucidate the functional contribution of PLK1 to smooth muscle contraction.

To inhibit PLK1 activity, we used volasertib, an ATP-competitive kinase inhibitor of the dihydropteridinone class. Volasertib potently inhibits PLK1 (IC_50_ = 0.87 nM) with 6- and 65-fold selectivity over PLK2 (IC_50_ = 5 nM) and PLK3 (IC_50_ = 56 nM), respectively. In contrast, assays conducted with a panel of more than 50 other kinases revealed no inhibitory activity, even at concentrations up to 10 μmol/L [18]. We selected concentrations of 100 nM and 1 µM volasertib, as this range ensures effective PLK1 inhibition while minimizing off-target effects. At 100 nM, Volasertib inhibits PLK1 activity, disrupting mitotic spindle formation and inducing cell-cycle arrest in M phase [33], while studies in airway smooth muscle indicate that relaxant effects occur at approximately 1 µM [7]. Thus, the 100 nM and 1 µM concentrations represent a balance between mechanistic relevance and the minimization of non-specific kinase inhibition, as broader kinase inhibition has been reported at concentrations above 10 µM [18,33]. It is important to note that the vascular experiments were conducted in Krebs buffer with a pH of 7.2, which represents a mildly acidic but physiologically acceptable environment. This condition was consistently maintained across all experimental groups.

The contractility of penile smooth muscle cells is controlled by several neurotransmitters and locally generated vasoactive chemicals, such as norepinephrine, Ang II, and endothelin-1, which induce contraction [34]. Moreover, penile arteries and veins, and cavernosal smooth muscle receive an abundant adrenergic innervation. It is widely acknowledged that the penis is maintained in the flaccid condition primarily through a tonic activity of the sympathetic nerves. Then, increased adrenergic response in the penis can compromise penile tumescence [35]. Given that α1-adrenergic receptors are highly expressed in vascular smooth muscle, their activation plays a critical role in inducing vascular contraction and elevating blood pressure [36]. PE is a sympathomimetic drug [37] that exerts its effects predominantly through direct stimulation of these receptors [38]. In this study, the effect of volasertib on the contraction response to PE was evaluated. Contraction mediated by PE showed a similar magnitude in the presence or absence of volasertib. Thus, volasertib does not appear to affect contraction induced by α1 adrenergic receptor activation in the arteries and CC, unlike in the human prostate, where α 1-adrenergic smooth muscle contraction can be inhibited by PLK inhibitors [9]. These results suggest that PLK1 activity may be differentially regulated across tissues and species.

Another contractile agent used in the arteries was the thromboxane A2 analog U46619. The actions of thromboxane A2 are mediated by the thromboxane-prostanoid receptor, a GPCR, which activates phospholipase C via Gαq. This is followed by an increase in intracellular free calcium concentration by activating inositol-3-phosphate-sensitive receptors and sensitizes the contractile machinery for Ca^2+^ [39]. Additionally, tyrosine kinase can be involved in Ca^2+^ influx stimulated by U46619 in rat dorsal penile arteries [40]. In the present study, volasertib did not reduce U46619-induced contraction in PA and SMA, suggesting that PLK1 activation is not involved in U46619-induced contraction. This finding is consistent with a previous study showing that U46619-induced contraction was unaffected by PLK inhibition in the human prostate [9].

Ang II is a potent vasoconstrictor and the primary effector of the renin-angiotensin system, which regulates essential physiological processes [41,42]. Ang II exerts its effects through two GPCRs: angiotensin type 1 receptor (AT1R) and angiotensin type 2 receptor (AT2R) [43]. Notably, physiologically relevant levels of Ang II are produced and secreted by the corpus cavernosum [44], with Ang II levels in cavernous blood reported to be 30% higher than in systemic blood [45]. Ang II plays a crucial role in initiating penile detumescence, suggesting a close connection between the renin-angiotensin system and the regulation of male reproductive function [46]. Additionally, elevated Ang II levels contribute to the development of conditions such as hypertension, atherosclerosis, cardiac hypertrophy, and diabetes by promoting vasoconstriction, endothelial dysfunction, vascular remodeling, and insulin resistance [41,42].

Becker and colleagues demonstrated that men with organic erectile dysfunction exhibit elevated levels of Ang II, suggesting that this peptide may play a significant role in the pathogenesis of erectile dysfunction [46]. In Ang II-infused hypertensive rats, NADPH oxidase activation has been identified as a key contributor to erectile dysfunction [47]. Notably, volasertib, at concentrations of 100 nM and 1 µM, reduced Ang II-induced contractions in both PA and SMA. While volasertib effectively reduced Ang II-induced contractions in both arteries, no such decrease in contraction was observed when the arteries were contracted with PE or U46619. This suggests that the inhibitory effects of volasertib on Ang II-induced contraction may be specific to the PLK1 signaling pathway activated by Ang II, rather than a general effect on smooth muscle contractility. The lack of a similar reduction in contraction induced by PE and U46619 implies that PLK1 may play a more prominent role in the Ang II-mediated contractile pathway. These findings align with a previous study by Cárcer and colleagues, which reported that contractile responses to Ang II were reduced in the aortas of PLK1 knockout mice and in mesenteric and aortic arteries treated with the PLK inhibitor BI2536 [19]. Under our experimental conditions, we did not investigate the effect of volasertib on Ang II-induced contractions in the CC, because no significant contraction was observed in response to Ang II administration in control conditions. This finding contrasts with reports in other species, such as human [48], canine [49], and rabbit [50] CC, where Ang II has been shown to evoke AT_1_ receptor–mediated contractions. In rats, the available evidence is limited and inconsistent, with one study demonstrating Ang II–induced contraction in Sprague-Dawley rat CC [51], whereas another reported no contractile effect in Wistar-Kyoto rats [52]. In mice, no studies have shown that Ang II alone induces contraction in isolated CC strips, although increased contractility to PE has been described in Ang II–infused mice [53]. These differences likely reflect interspecies variations in the local renin–angiotensin system, including receptor expression, distribution, and downstream signaling pathways, as well as methodological factors such as tissue preparation, pre-contraction level, and experimental conditions.

Although our data suggest a role for PLK1 in vascular smooth muscle Ang II-induced contraction, the underlying activation mechanisms require further investigation. Previous study demonstrated that in response to contractile stimulation, PLK1 is phosphorylated at Thr-210, and then mediates vimentin phosphorylation at Ser-56 [7]. Vimentin is a cytoskeletal intermediate filament protein that plays a crucial role in regulating the mechanical properties of smooth muscle, including tension and contractility, by stabilizing intercellular and intracellular mechanical transmission [54]. Furthermore, previous research suggests that Ang II stimulates vimentin phosphorylation in vascular SMC through a calcium-dependent mechanism that does not involve protein kinase C [55]. These findings imply that PLK1 may regulate arterial contraction by facilitating vimentin phosphorylation as part of the Ang II-triggered signaling cascade. PLK1 can also regulate Ang II–induced activation of RhoA and actomyosin dynamics in vascular SMCs independently of mitosis [19]. Our study highlights that PLK1 activation potentially influences Ang II-induced contraction in both PA and SMA, offering new insights into its role in vascular regulation. Further research is needed to elucidate the mechanisms underlying this relationship and its implications for vascular function and pathology.

Beyond its role in vimentin phosphorylation, PLK1 also regulates Ang II–induced activation of RhoA and actomyosin dynamics in vascular SMCs, independent of its mitotic functions. Specifically, in VSMCs, AngII stimulation promotes stress fiber formation by activating RhoA and inducing the phosphorylation of myosin light chain (MLC). The loss of Plk1 disrupted this process, resulting in reduced phosphorylation of MLC and Mypt1, as well as impaired RhoA activation. Plk1 deletion in VSMCs led to defective actomyosin cytoskeletal remodeling in a RhoA-dependent manner. However, restoring RhoA activity through the expression of a constitutively active form successfully rescued these defects, indicating that Plk1 is essential for regulating RhoA signaling in VSMCs. These findings highlight the critical role of the Plk1–RhoA pathway in modulating vascular contractility [19].

Additionally, PLK1 has been implicated in the AMP-activated protein kinase (AMPK)-mediated activation of myosin regulatory light chain (MRLC), revealing molecular crosstalk between PLK1 and MRLC [56]. This interaction is significant because the phosphorylation of MRLC is a key regulatory event that influences various cellular processes, including muscle contraction, mitosis, and cytokinesis [57]. Specifically, phosphorylation of the myosin MRLC plays a critical role in smooth muscle contraction by promoting the cycling of actin-myosin cross-bridges [58]. This suggests that PLK1 could play a more direct role in regulating smooth muscle contraction by modulating MRLC phosphorylation.

It is important to note that there is no information in the literature regarding a potential direct interaction between the AT_1_ receptor and volasertib, including whether volasertib could act as an AT_1_ receptor antagonist. While further research would be required to definitively rule out this possibility, previous work in smooth muscle–specific PLK1 knockout mice (Plk1^Δ/Δ(SM)^) demonstrated a markedly impaired contractile response of aortic rings to AngII, indicating that PLK1 is required for AngII–mediated contraction [19]. This genetic evidence strongly supports the interpretation that the inhibitory effect of volasertib on AngII responses is mediated through its intended inhibition of PLK1 rather than through off-target antagonism of the AT_1_ receptor.

5-HT is a monoamine transmitter that plays a variety of biological roles in both peripheral organs and the central nervous system. Most of the 5-HT receptors discovered are G-protein-coupled receptors, with the exception of the 5-HT_3_ receptor [59]. 5-HT can induce contraction of the cavernosal smooth muscle, primarily through the involvement of the 5-HT_1A_, 5-HT_1B_, and 5-HT_2A_ receptors [60]. It has been shown that 5-HT plays a role in maintaining penile flaccidness and promoting detumescence [61]. Some studies have reported 5-HT-induced contractions in isolated human [61,62] and rabbit CC [63]. Thus, 5-HT is considered an endogenous contractile factor of the CC. Moreover, 5-HT-induced contraction in human CC is largely mediated via the RhoA/Rho-kinase pathway [62]. Similarly, our results show that 5-HT caused a concentration-dependent contraction in mice CC. In CC strips preincubated with volasertib, the maximal contraction induced by 5-HT and the pEC50 values were similar to the control. These findings suggest that PLK1 activation is not involved in 5-HT-induced contractions in mice CC.

In experiments evaluating noradrenergic contractions, the CC was incubated with the NOS inhibitor L-NAME and the nonselective muscarinic receptor antagonist atropine, allowing assessment of electrical field stimulation-induced contractions primarily via adrenergic nerve fiber activation. PLK1 inhibition reduced contractions induced by EFS at 2 and 4 Hz, suggesting that PLK1 contributes to the regulation of adrenergic-mediated contractile responses in the CC. However, at higher frequencies (8 and 16 Hz), the responses were comparable to the control. At lower stimulation frequencies, neurotransmitter release and the resulting contractile response are submaximal and remain more susceptible to intracellular modulation. In contrast, higher frequencies evoke greater neurotransmitter release, leading to near-maximal activation of contractile mechanisms; consequently, the stronger neurogenic contractile drive at these frequencies may mask the inhibitory influence of PLK1.

We also observed discrepant results when comparing the effects of volasertib on PE-induced and electrical field stimulation-induced contractions in the CC. PE directly activates α_1_-adrenergic receptors on SMC, leading to contraction. In contrast, electrical field stimulation induces contraction through the activation of adrenergic nerve fibers and purinergic innervation, triggering the release of endogenous norepinephrine and ATP, respectively [64]. This process involves more complex signaling pathways, including α_1_-adrenergic receptor activation, as well as additional factors related to nerve transmission and the surrounding smooth muscle environment. Thus, while PE acts through a direct receptor-mediated mechanism, electrical field stimulation-induced contraction depends on a multifaceted signaling cascade that integrates both neuronal and receptor-level interactions. This distinction may explain the different responses to PLK1 inhibition, highlighting the need for further investigation to fully elucidate the underlying mechanisms.

Evidence indicates that Ang II stimulates vimentin phosphorylation via a calcium-dependent pathway in cultured vascular SMC [55]. We hypothesize that Ang II promotes the phosphorylation of cytoskeletal proteins, potentially through interaction with PLK1, which regulates various cellular processes by phosphorylating a diverse array of substrates, including cytoskeletal proteins.

PLK-1 plays a crucial role in amplifying Ang II-induced arterial contraction by enhancing RhoA signaling and actomyosin interactions, thereby maintaining proper vascular tone regulation. Through its activation of RhoA, PLK-1 promotes downstream signaling that facilitates actomyosin contraction, ensuring effective vasoconstriction [19]. However, overactivation of the PLK-1 → RhoA → actomyosin pathway may contribute to pathological vascular remodeling and hypertension by increasing vascular resistance and promoting excessive smooth muscle contraction. These findings highlight PLK-1 as a potential therapeutic target for conditions characterized by abnormal vascular tone and remodeling.

It is important to note that assessing PLK1 phosphorylation in intact vessels exposed to Ang II remains technically challenging. In wire myograph experiments, Ang II evokes a rapid, transient contraction that peaks within ~1–2 min and then declines toward baseline. PLK1 phosphorylation is likely even more transient, potentially occurring and returning to baseline within seconds. The combination of a short-lived contractile response and an even briefer biochemical signal makes it difficult to capture the precise phosphorylation peak under these conditions.

While the present results provide valuable insights into a novel signaling molecule involved in smooth muscle contractility, further investigation is needed to fully understand how PLK1 contributes to this process. Additional studies are also required to determine whether increased PLK1 activation plays a role in the development of sexual dysfunction in conditions such as hypertension and diabetes. Moreover, the limited research on PLK1 role in SMC contraction prevents direct comparisons with other studies on this topic. It is also important to note that, while volasertib was used to inhibit PLK1 activity, this compound can inhibit PLK2 and PLK3 at the concentrations used. Therefore, the potential involvement of PLK2 and PLK3 in the observed effects cannot be excluded. Future studies utilizing genetic knockdown or knockout models will be essential to confirm the isoform-specific roles of PLK family members in regulating smooth muscle contractility. Finally, although the mouse is a valuable model for studying erectile physiology, species-specific differences in vascular and neurovascular regulation limit the direct extrapolation of these findings to humans [65,66]. Accordingly, future studies using human tissues will be essential to validate the role of PLK1 in human vascular and erectile physiology.

Our findings raise translational considerations for oncology clinical trial patients receiving PLK1 inhibitors, in whom vascular and erectile functions should be monitored. More broadly, the emerging roles of PLK1 in vascular and cavernosal smooth muscle suggest a targetable regulatory pathway relevant to disorders of smooth muscle tone.

## 5. Conclusions

In conclusion, our findings suggest that PLK1 acts as a regulator of specific contractile stimuli involved in both vascular and cavernosal smooth muscle contractility. The observed effects of PLK1 inhibition highlight its contribution to contractile signaling activated by neurogenic and Ang II–dependent pathways. These findings raise important translational implications, as PLK1-targeted therapies currently under evaluation in oncology could potentially influence vascular and erectile function. Further studies are warranted to determine the systemic impact of PLK1 inhibition on vascular health and erectile physiology in clinical settings.

## Data Availability

The data that support the findings of this study are available from the corresponding author upon reasonable request.

## Supplementary Materials

The following supporting information can be downloaded at: https://www.mdpi.com/article/10.3390/cells14211741/s1. Figure S1. Contractile responses to electrical field stimulation (EFS) in mouse corpus cavernosum under control conditions and after incubation with 50% DMSO. Figure S2. Detection of PLK1 protein in small mesenteric arteries (SMA). Western blot analysis showing PLK1 expression in SMA using anti-PLK1 antibody (ab189139). Figure S3. Concentration–response curves to Volasertib in phenylephrine (PE)-precontracted SMA and pudendal arteries (PA) from C57BL/6J mice.
