# Supplementary material for: Polo-like Kinase 1 Activation Regulates Angiotensin II-Induced Contraction in Pudendal and Small Mesenteric Arteries from Mice
Source: Cells. 2025 Nov 6;14(21):1741. doi: 10.3390/cells14211741 (PMC12610839; doi:10.3390/cells14211741)
Supplement: Supplementary file 1 [file cells-14-01741-s001.zip › cells-3874297-supplementary.pdf]

Polo-like Kinase 1 Activation Regulates Angiotensin II-Induced Contraction in Pudendal and Small Mesenteric Arteries from Mice

Raiana Anjos Moraes, Olufunke O. Arishe, James Pratt, Stephanie Wilczynski, Rinaldo Rodrigues dos Passos, Diana L. Silva-Velasco, Tiago Tomazini Gonçalves, Tianxin Zhang, Darizy Flavia Silva, R. Clinton Webb and Fernanda Priviero

SUPPLEMENTARY FIGURES

To ensure that repeated electrical field stimulation in the same strip did not affect tissue responsiveness, we previously conducted control experiments with two consecutive electrical field stimulation applications, with vehicle treatment in between, and observed no significant differences (Figure S1).

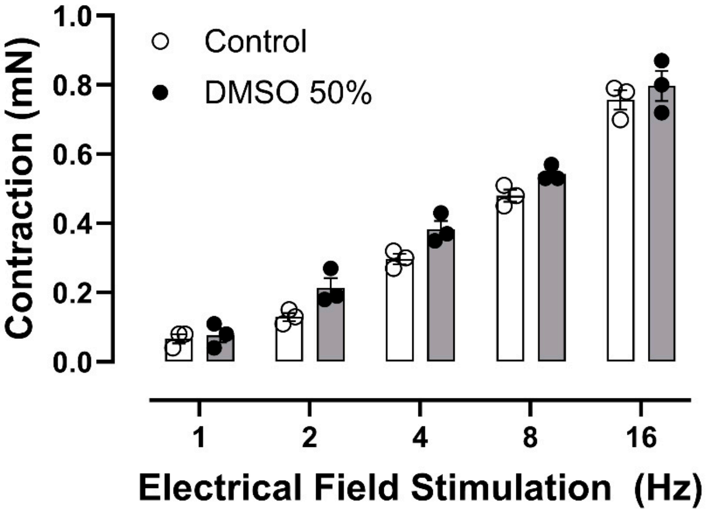

Figure S1. Contractile responses to electrical field stimulation (EFS) in mouse corpus cavernosum under control conditions and after incubation with 50% DMSO. EFS (1–16 Hz) evoked frequency-dependent contractions that were not significantly affected by DMSO. Data are presented as mean ± SEM; n = 3 per group.

In addition, we performed Western blot analysis on small mesenteric arteries (SMA) using a different anti-PLK1 antibody (ab189139), confirming PLK1 expression

in this tissue. A band was detected at approximately 75 kDa, consistent with the expected molecular weight of PLK1 (~68 kDa; Figure S2).

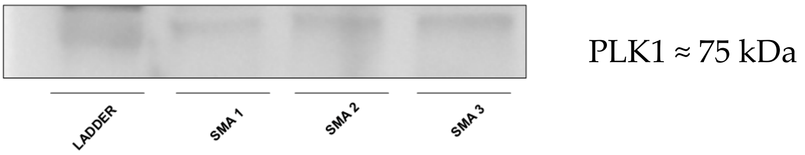

Figure S2. Detection of PLK1 protein in small mesenteric arteries (SMA). Western blot analysis showing PLK1 expression in SMA using anti-PLK1 antibody (ab189139).

In our study, concentration–response experiments were performed in small mesenteric and pudendal arteries precontracted with phenylephrine (Figure S3). It was not feasible to generate a similar curve in arteries precontracted with angiotensin II (Ang II) due to the transient nature of Ang II-induced contraction. It is also important to note that vascular responses to Volasertib may vary depending on the contractile agonist (e.g., phenylephrine vs. Ang II), likely due to differences in the intracellular signaling pathways activated by these stimuli.

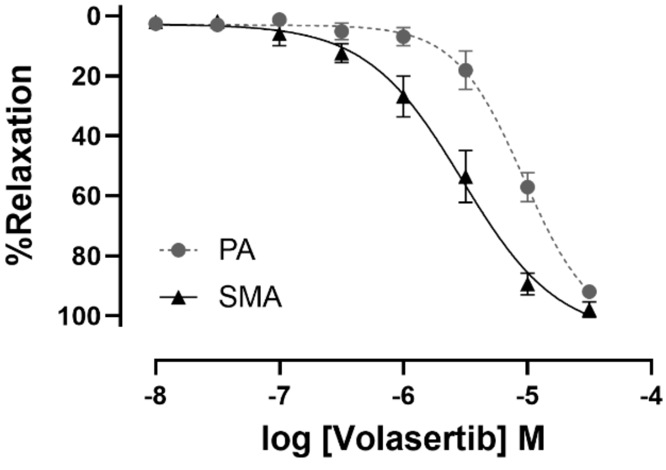

Figure S3. Concentration–response curves to Volasertib in phenylephrine (PE)-precontracted small mesenteric arteries (SMA) and pudendal arteries (PA) from C57BL/6J mice. Data are presented as mean ± SEM; n = 4 for each group.
